# Identification of Novel Pax8 Targets in FRTL-5 Thyroid Cells by Gene Silencing and Expression Microarray Analysis

**DOI:** 10.1371/journal.pone.0025162

**Published:** 2011-09-23

**Authors:** Tina Di Palma, Anna Conti, Tiziana de Cristofaro, Serena Scala, Lucio Nitsch, Mariastella Zannini

**Affiliations:** 1 Institute of Experimental Endocrinology and Oncology ‘G. Salvatore’ (IEOS), National Research Council, Naples, Italy; 2 Department of Cellular and Molecular Biology and Pathology, University of Naples Federico II, Naples, Italy; Cardiff University, United Kingdom

## Abstract

**Background:**

The differentiation program of thyroid follicular cells (TFCs), by far the most abundant cell population of the thyroid gland, relies on the interplay between sequence-specific transcription factors and transcriptional coregulators with the basal transcriptional machinery of the cell. However, the molecular mechanisms leading to the fully differentiated thyrocyte are still the object of intense study. The transcription factor Pax8, a member of the *Paired-box* gene family, has been demonstrated to be a critical regulator required for proper development and differentiation of thyroid follicular cells. Despite being Pax8 well-characterized with respect to its role in regulating genes involved in thyroid differentiation, genomics approaches aiming at the identification of additional Pax8 targets are lacking and the biological pathways controlled by this transcription factor are largely unknown.

**Methodology/Principal Findings:**

To identify unique downstream targets of Pax8, we investigated the genome-wide effect of Pax8 silencing comparing the transcriptome of silenced versus normal differentiated FRTL-5 thyroid cells. In total, 2815 genes were found modulated 72 h after Pax8 RNAi, induced or repressed. Genes previously reported to be regulated by Pax8 in FRTL-5 cells were confirmed. In addition, novel targets genes involved in functional processes such as DNA replication, anion transport, kinase activity, apoptosis and cellular processes were newly identified. Transcriptome analysis highlighted that Pax8 is a key molecule for thyroid morphogenesis and differentiation.

**Conclusions/Significance:**

This is the first large-scale study aimed at the identification of new genes regulated by Pax8, a master regulator of thyroid development and differentiation. The biological pathways and target genes controlled by Pax8 will have considerable importance to understand thyroid disease progression as well as to set up novel therapeutic strategies.

## Introduction

The thyroid is mainly composed of highly differentiated epithelial cells known as thyroid follicular cells (TFC), that are devoted to the production and export of thyroid hormones, such as thyroxine (T4) and triodothyronine (T3), essential for growth, development and survival. The differentiation program of thyroid follicular cells relies on the interplay between transcription factors and transcriptional coregulators with the basal transcriptional machinery. The simultaneous and orchestrated expression of these proteins plays a pivotal role in the control and maintenance of the differentiated phenotype, determining the expression of a set of thyroid-specific genes. Some of these genes, such as thyroglobulin (Tg) and thyroperoxidase (TPO), are only expressed in thyroid cells; others, such as sodium-iodide symporter (NIS), thyrotropin receptor (TSHr), pendrin, Hex and thyroid oxidase (THOX) are much more abundant in the thyroid as compared to other tissues [1], [2].

Among the transcription factors involved in the expression of thyroid-specific genes there is Pax8, a member of the Pax (Paired-box) gene family [3]. Like other members of the family, Pax8 is thought to orchestrate the patterns of gene expression in specific cells during organ development. During thyroid development, Pax8 is expressed upon transition from undifferentiated endoderm cells to thyroid follicular fated cells in the thyroid anlage and continues to be expressed throughout development and in the adult gland [1]. It has been clearly demonstrated that Pax8 is necessary for the expression of thyroid-specific genes, like thyroglobulin (Tg), thyroperoxidase (TPO), and sodium/iodide symporter (NIS) essential for the synthesis of active thyroid hormones [4], [5], [6]. In addition, in Pax8 knockout mice the thyroid gland is barely visible and lacks the follicular cells [7]. The critical role exerted by Pax8 in TFC differentiation has been demonstrated also in cell culture systems. For example, in thyroid cells expressing the polyoma virus middle T antigen (PCPy cells), loss of Pax8 expression results in loss of the thyroid-differentiated phenotype, measured as the expression of Tg, TPO and NIS genes. Re-introduction of Pax8 in PCPy cells is sufficient to re-activate the expression of the endogenous Tg, TPO and NIS genes [5]. Given the pivotal role played by Pax8 in thyrocyte differentiation, in the past years many studies have been focused on the molecular mechanisms by which Pax8 modulates thyroid gene expression. Recently, it has been demonstrated that Pax8 biochemically interacts with Titf1/Nkx2.1 and the interaction between the two factors has an important functional relevance since they strongly synergize in the transcriptional activation of thyroid-specific genes [8]. Moreover, by using different experimental strategies, two interesting partners of Pax8, PARP-1 and TAZ, have been identified [9], [10].

PAX8 mutations and inactivation are implicated in various thyroid conditions. Congenital hypothyroidism is caused by several genetic defects and among these there are mutations in the PAX8 gene [1], [11], [12]. In addition to hypothyroidism, PAX8 plays a role also in the progression of follicular thyroid carcinomas and adenomas [13], [14]. In tissues other than the thyroid, PAX8 was found overexpressed in epithelial ovarian cancer [15] and it has been hypothesized that PAX8 maybe an important regulator of telomerase activity and cell survival in some gliomas [16].

Although the crucial role of Pax8 during development and differentiation of thyroid cells has been well established, very little is known about Pax8 target genes in these cells. Therefore, we performed a genome-wide screening to identify novel Pax8 targets, thus acquiring relevant information on molecular events controlling thyroid function.

## Results

### Gene expression profile changes upon knock-down of Pax8

**Figure 1:**
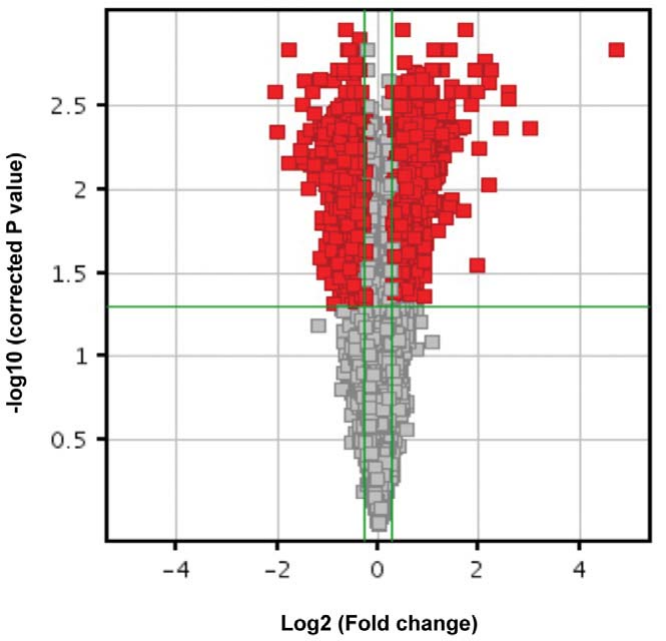
Volcano plot representation of dysregulated genes. One way ANOVA test with Benjamini and Hochberg false discovery rate (FDR) correction was applied to the comparison between the conditions (siPax8 *vs.* controls), using a 0.05 threshold for statistical significance. The negative log10-transformed p-values are plotted against the log ratios (log2 fold change) between conditions. Red dots represent differentially expressed genes that show both large fold changes and high statistical significance. Gray dots represent all the other genes.

To identify genes regulated by Pax8 in differentiated thyroid cells, FRTL-5 cells were transfected with siGENOME Pax8 siRNA (experimental) or siGENOME Non-Targeting siRNA (control), a scrambled sequence that does not show homology with any rat gene. RNAs extracted from the cells 72 h after transfection were hybridized to the Affymetrix GeneChip® Rat Gene 1.0 ST, a high density oligonucleotide array capable to analyze 27,342 well-annotated rat genes. Data from three independent silencing experiments were analyzed unraveling 2815 genes differentially expressed between silenced cells and scramble controls, with fold change greater than 1.2 (FDR p <0.05) (see Table S1). Of these, 1421 genes were down-regulated and 1394 genes were up-regulated as shown by the Volcano Plot representation (Figure 1). As expected, Pax8 was differentially expressed in the two samples (fold decrease = 1,7324) as were also several already known Pax8 target genes (Table 1).

**Table 1:** Summary of known Pax8 target genes.

Gene symbol	Gene description	Fold change	Reference
Tg	thyroglobulin	−3,35028	[8], [24]
Slc5a5	solute carrier family 5 (sodium iodide symporter), member	−2,07442	[6]
TPO	thyroperoxidase	−1,94253	[8]
Hhex	hematopoietically expressed homeobox	−1,7037	[4]
Foxe1	forkhead box E1 (thyroid transcription factor 2)	−1,48761	[24]
Duox2	dual oxidase 2	−1,15531	[24]

**Figure 2:**
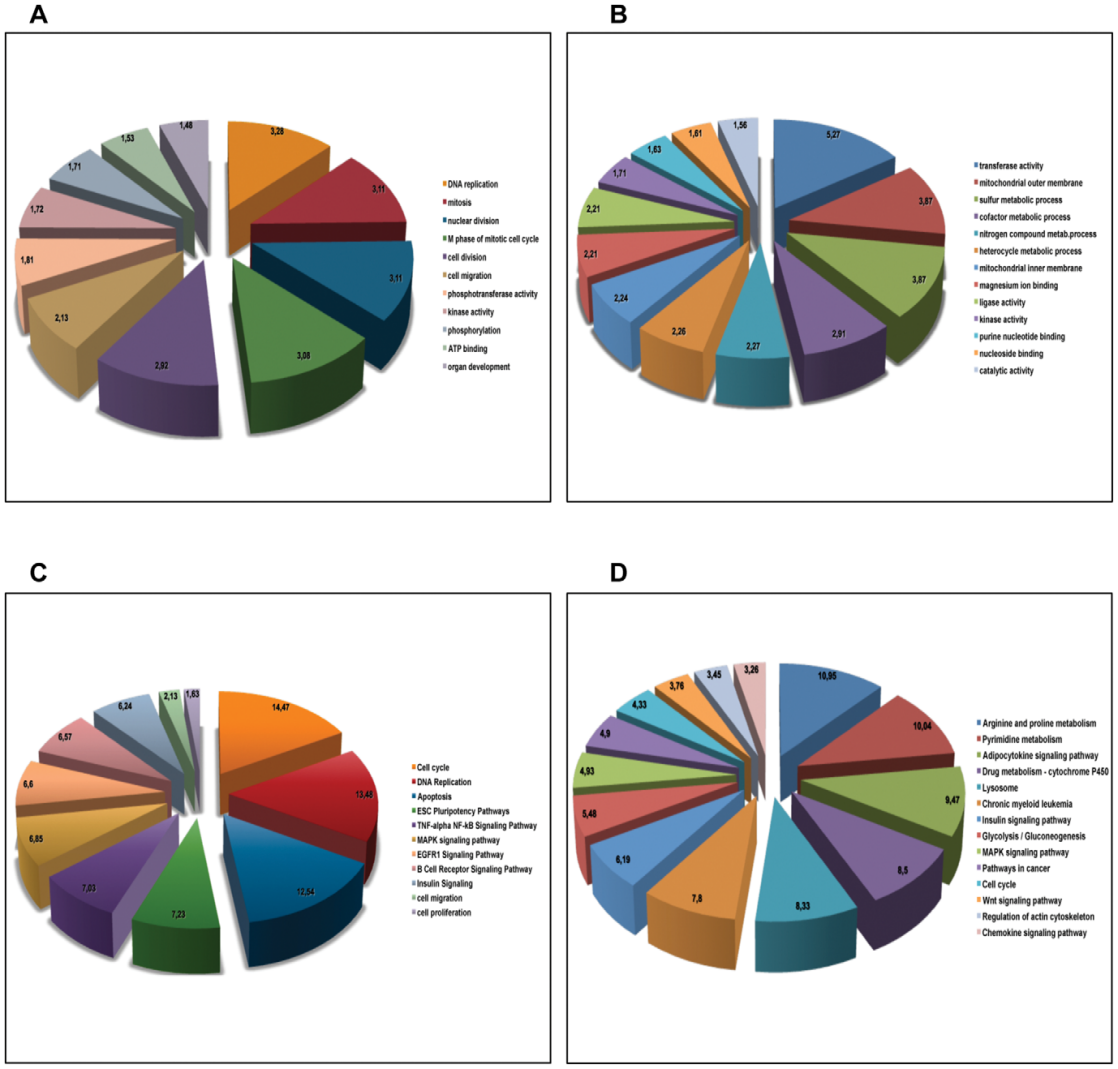
Functional class scoring of the differentially expressed genes. Gene ontology (GO) and pathway functional class scoring have been performed using the Gene Set Analysis Toolkit V2 (http://bioinfo.vanderbilt.edu/webgestalt). **A**) GO categories enriched for genes down-regulated after Pax8 silencing. **B**) GO categories enriched for genes up-regulated after Pax8 silencing. **C**) KEGG (Kyoto Encyclopedia of Genes and Genomes) pathways enriched for genes down-regulated after Pax8 silencing. **D**) KEGG pathways enriched for genes up-regulated after Pax8 silencing.

Gene ontology (GO) functional class scoring of the differentially expressed genes demonstrated that the most affected categories were: DNA replication, mitosis, nuclear division, M phase of mitotic cell cycle, cell migration for the down-regulated genes (Figure 2A) and transferase activity, mitochondrial outer membrane, sulfur metabolic process, cofactor metabolic process for the up-regulated genes (Figure 2B). Accordingly, the most affected pathways were cell cycle, DNA replication, apoptosis for the downregulated genes (Figure 2C) and arginine and proline metabolism, pyrimidine metabolism, adipocytokine signaling pathway, drug metabolism, lysosome for the upregulated ones (Figure 2D).

**Figure 3:**
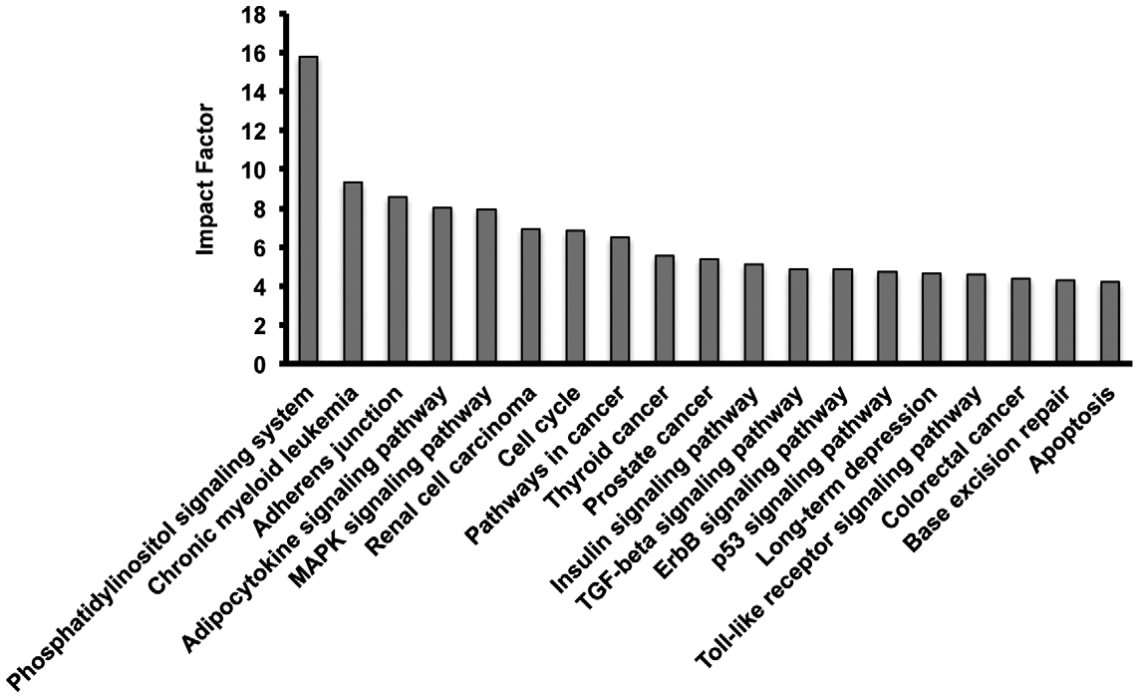
Pathway analysis of genes differentially expressed in siPax8 *vs.* controls. Pathway-Express software was used to identify the pathways most affected by the gene dysregulation. The histograms represent affected pathways ranked according to the impact factor, an index that measures the pathway perturbation [56].

Pathway express analysis of all the dysregulated genes demonstrated that 19 pathways (Figure 3), including phosphatidylinositol signaling, adherens junction, MAPK signaling, cell cycle, thyroid cancer as well as other cancer pathways, insulin signaling, p53 signaling and apoptosis, were highly perturbed upon silencing of Pax8.

Since the goal of the study was the identification of novel Pax8 targets in FRTL-5 cells, a strategy was developed to reduce the number of false positive targets filtering data according to tissue specificity and Transcription Factor Binding Sites (TFBSs) conservation. Differentially expressed genes were filtered according to their thyroid-enriched expression using the BioGps gene annotation portal and the GeneHub Gepis bioinformatics tool. To identify Pax8 direct targets, the filtered genes were analyzed for the presence in their 5’-flanking regions of Pax8 binding consensus sequences and were ranked according to their affinity score to PAX8_01 and PAX8_B matrices ≥1.4 using the PASTAA method [17]. This cutoff level was chosen in order to include all the well-known Pax8 targets.

A total of 276 genes, 134 down-regulated and 142 up-regulated upon Pax8 silencing, were selected (Table S2).

### Validation of a set of genes dysregulated by Pax8 silencing

**Figure 4:**
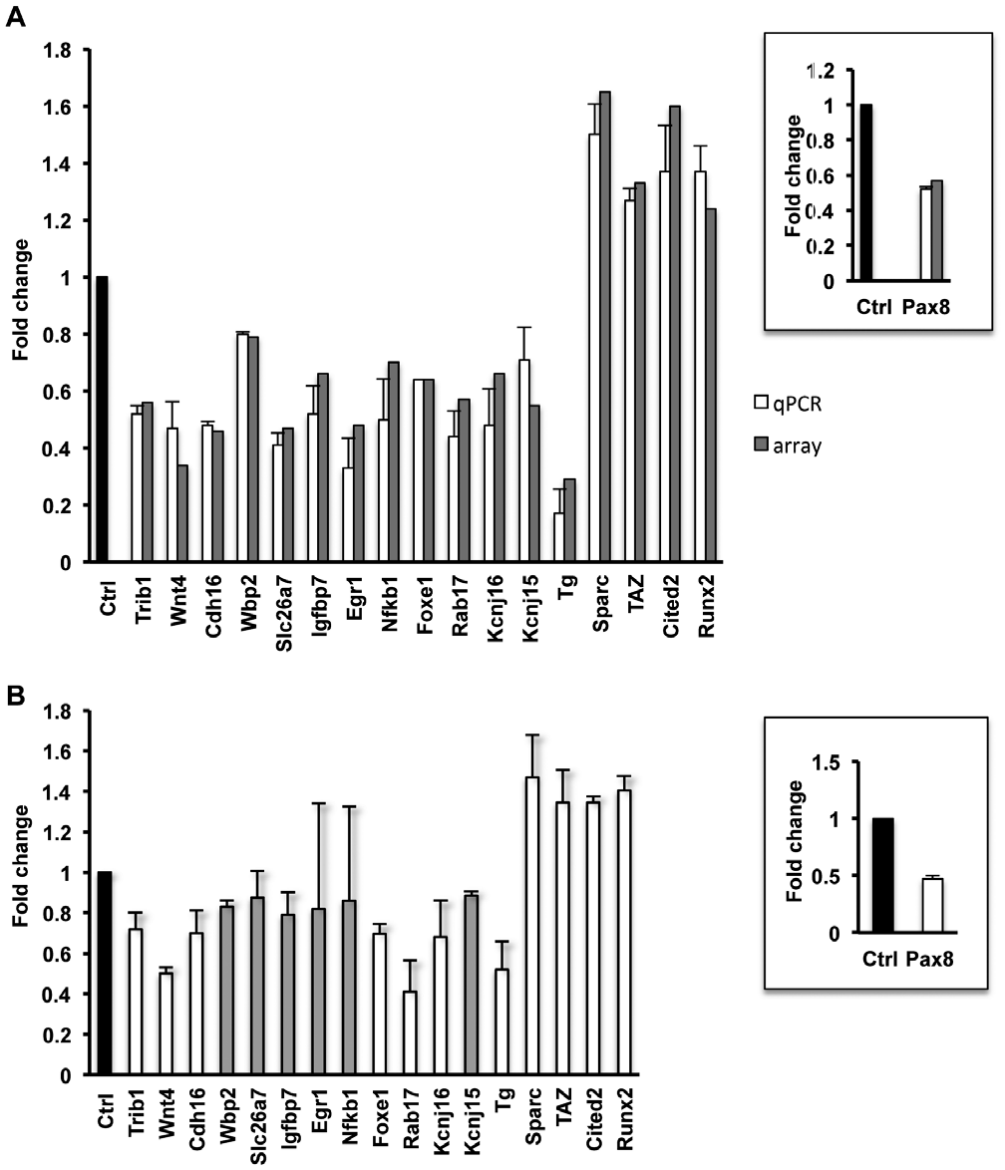
Validation of the microarray results by qRT-PCR. Transcript abundance of 17 selected genes was measured by qRT-PCR. Relative gene expression in siPax8 transfectants was calculated using non-targeting siRNA transfectant RNA (Ctrl) as reference. **A**) Differential gene expression in siPax8 vs Ctrl cells, 72 h after Pax8 silencing: comparison between qRT-PCR and array data validates array results with a correlation coefficient r > 0.80. **B)** Differential gene expression in siPax8 vs Ctrl cells, 24 h after Pax8 silencing. The experiment was repeated three times on different biological replicates. White bars indicate differentially expressed genes with absolute fold change>1.2 and high statistical significance (p<0.05 for t-test). These genes are presumably direct Pax8 targets. In both panels, the fold change of Pax8 expression level is shown in the insert.

To validate the expression data obtained by the microarray analysis, we performed qRT-PCR for 17 genes, 13 down-regulated and 4 up-regulated, that were randomly chosen from the ranked lists of Pax8 putative targets obtained by the PASTAA analysis (Table 2). qRT-PCR on RNA samples prepared 72 h after Pax8 siRNA transfection confirmed the microarray results for all selected genes (Figure 4A). In particular, we identified *Trib1, Wnt4, Cdh16, Wbp2, Slc26a7, Igfbp7, Egr1, Nfkb1, Rab17, Kcnj16, Kcnj15* as genes positively regulated by Pax8; *Sparc, Cited2, TAZ* and *Runx2* as genes negatively regulated by Pax8. Additionally, the down-regulation of Tg and Foxe1 genes, both known Pax8 targets [6], [18] was confirmed thus indicating that the experiment was well performed and the expression data are reliable.

**Table 2:** Genes differentially expressed in siPax8 cells versus siNon-targeting cells selected by the PASTAA analysis.

Common	Synonyms	ENTREZ Gene ID	Affinity Score
*Down-regulated genes*			
**Rab17**	**RAB17, member RAS oncogene family**	**503269**	**2.07**
Slc26a7	Solute carrier family 26, member 7	297910	**2.05**
**Trib1**	**Tribbles homolog 1 (Drosophila)**	**78969**	**1.98**
**Wnt4**	**Wingless-type MMTV integration site family, member 4**	**84426**	**1.93**
**Tg**	**Thyroglobulin**	**24826**	**1.92**
**Kcnj16**	**Potassium inwardly-rectifying channel, subfamily J, member 16**	**29719**	**1.88**
Kcnj15	Potassium inwardly-rectifying channel, subfamily J, member 15	170847	1,84
Igfbp7	Insulin-like growth factor binding protein 7	289560	1.79
Nfkb1	Nuclear factor of kappa light polypeptide gene enhancer in B-cells 1	81736	1.79
Egr1	Early growth response 1	24330	1.78
**Cdh16**	**Cadherin 16, KSP-cadherin**	**307614**	**1.71**
Wbp2	WW domain binding protein 2	192645	1.61
**Foxe1**	**Forkhead box E1 (thyroid transcription factor 2)**	**192274**	**1.40**
*Up-regulated genes*			
**Runx2**	**Runt-related transcription factor 2**	**367218**	**2.28**
**Cited2**	**Cbp/p300-interacting transactivator, with Glu/Asp-rich carboxy-terminal domain, 2**	**114490**	**1.95**
**TAZ**	**transcriptional co-activator with PDZ-binding motif**	**295062**	**1.93**
**Sparc**	**secreted protein, acidic, cysteine-rich glycoprotein**	**24791**	**1.68**

All the genes are modulated at least 1.3 fold with a *p*−value<0.05. We show 13 genes down-regulated and 4 genes up-regulated with affinity score to PAX8≥1.40, selected for further analysis. The genes reported in bold are the ones validated by qPCR after 24 h siRNA transfection.

To test whether Pax8 was directly involved in the transcriptional regulation of the validated genes, the effect of Pax8 silencing on their expression 24 h after siRNA transfection was evaluated. This time point was chosen because the half-life of the Pax8 protein was found to be approximately 16 h (our unpublished data). Eleven transcripts responded significantly to Pax8 silencing 24 h after siRNA transfection (Figure 4B), and these were *Trib1, Wnt4, Cdh16, Rab17, Kcnj16, Tg and Foxe1* which were downregulated and *Sparc, Cited2, TAZ* and *Runx2* which were upregulated.

**Figure 5:**
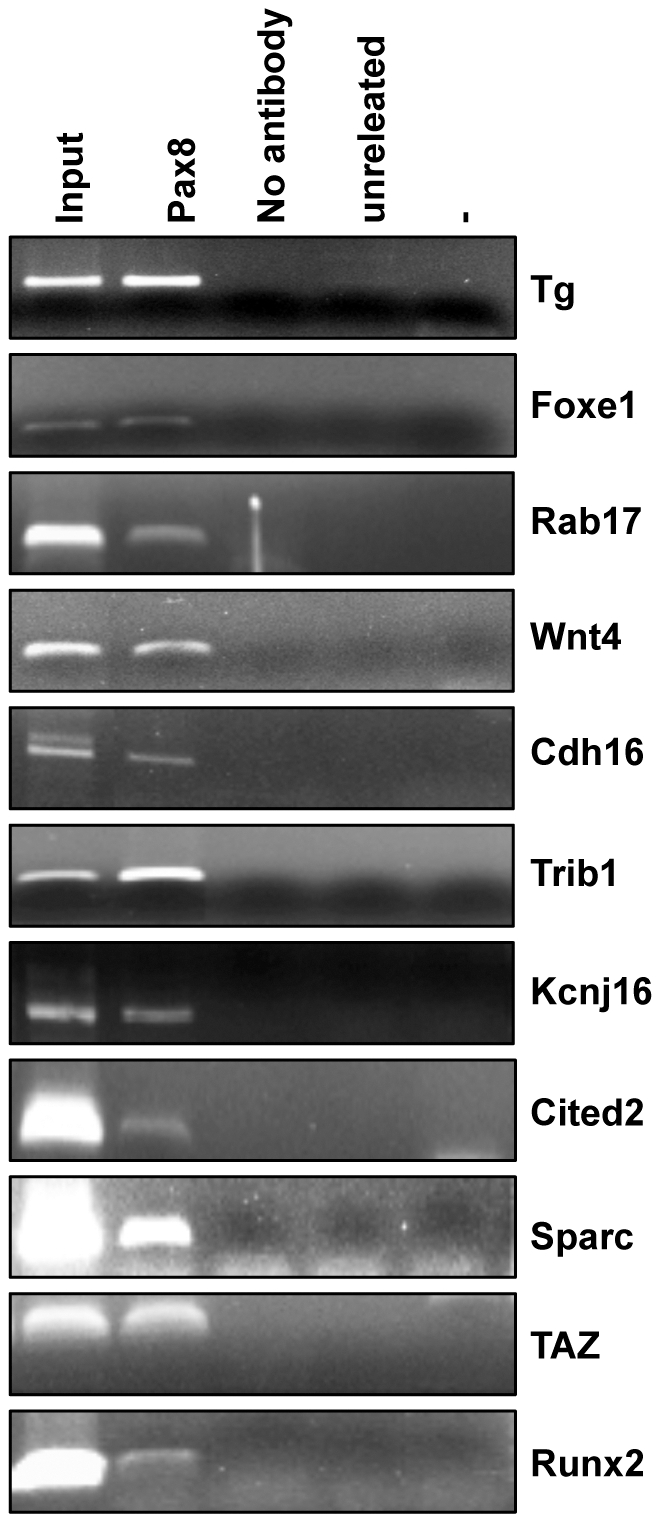
ChIP experiments for Pax8 binding to predicted targets. Chromatin Immunoprecipitation performed on FRTL-5 cells with Pax8 antibody followed by PCR analysis of 11 predicted Pax8 targets shows the binding *in vivo* of Pax8 to the 5’-flanking regions of these genes.

To unambiguously determine whether Pax8 regulated these genes by directly binding to their regulative genomic sequences, a computational analysis using the MatInspector Software 8.0 was performed. We searched for Pax8 binding sites in a region of about 3000 bp in their 5’-flanking region, and the analysis showed the presence of Pax8 consensus sequences in all the genomic regions analyzed. To confirm the predictions of the MatInspector analysis, chromatin immunoprecipitation (ChIP) assays on FRTL-5 cells was carried out. The cross-linked chromatin was immunoprecipitated using a polyclonal antibody against Pax8. As control, to rule out unspecific background of the ChIP assay, one reaction lacking the primary antibody was performed. For each gene, 2 or 3 pairs of oligonucleotides to amplify two or three genomics regions upstream the coding sequences were designed. The sequences of the oligonucleotides that showed positive results are reported in Table S3. In line with our previous results, we demonstrated the binding of Pax8 to the 5’-flanking region of all the genes modulated by Pax8 silencing already 24 h after siRNA transfection (Figure 5). Furthermore, in agreement with our published data [18], Pax8 antibody was able to immunoprecipitate the chromatin containing the Tg and Foxe1 gene promoters (Figure 5).

In conclusion, we identified new Pax8 binding sites in the 5’-flanking regions of *Trib1*, *Wnt4*, *Cdh16*, *Rab17*, *Kcnj16*, genes that are downregulated 24 h after Pax8 silencing, and of *Sparc*, *Cited2*, *TAZ*, *Runx2*, genes that are upregulated 24 h after Pax8 silencing, suggesting that these genes are direct downstream targets of this transcription factor.

## Discussion

It is well established that the transcription factor Pax8 plays a crucial role in thyroid phenotype determination, thyroid development and differentiation [3], [5]. In order to define the transcriptional network functionally regulated by Pax8 as well as infer its direct targets, we performed RNAi to knock-down Pax8 gene in FRTL-5 thyroid cells and we analyzed the gene expression profile by microarray analysis. The results obtained suggest that Pax8 regulates several pathways, mainly involved in the regulation of cell cycle, thyroid cancer and apoptosis (Figure 3), thus confirming that Pax8 is a master regulatory gene. Among the genes that we validated there are *Cited2, TAZ, Runx2, Trib1, Sparc, Wnt4, Rab17, Kcnj16 and Cdh16*. We have chosen these genes from both the downregulated and upregulated ranked lists of genes according to their expression in fetal or adult thyroid and predicted affinity of their 5’-flanking regions to Pax8 binding. Moreover, these genes are included in the most represented gene categories such as regulation of cell transcription, regulation of cell proliferation, organ development, signal transduction, cell migration and regulation of localization.

*Cited2, TAZ, Runx2* and *Trib1* belong to the categories of regulation of cell transcription and cell proliferation.

*Cited2* (CBP/p300 interacting coactivator with glutamic acid/aspartic acid-rich tail 2) is a bifunctional protein, critical for the regulation of various cellular processes, especially cell growth and oncogenesis. Initially described as a corepressor of hypoxia-inducing factor 1α (HIF1α) by competing for CBP/p300 binding [19], it also functions as a coactivator of AP-2 and PPARα and PPARγ by recruiting CBP/p300 [20], [21]. In the literature, different observations suggest that Cited2 may be a pro-apoptotic factor [22], but it may also have anti-apoptotic properties [23]. Thus, the transcriptional regulation of Cited2 by Pax8 could play an important role in the regulation of cell growth and survival.

*TAZ*, also known as *Wwtr1*, was recently identified as a transcriptional co-activator of various transcription factors, including Runx2/Cbfa1 [24], [25], the TEF-1 gene family [26], TBX5 [27], Pax3 [28], TTF-1 [29]. Recently, we have demonstrated that TAZ acts as a potent regulator of Pax8 and TTF-1 activity [9] and we have also reported that TAZ, already known to promote cell proliferation and to induce epithelial–mesenchymal transition [30], is overexpressed in papillary thyroid cancer [31]. Our findings here presented suggest that Pax8 could directly regulate TAZ expression, in addition to physically interact with it to control finely thyroid development and differentiation. It is worth to underline that TAZ plays a crucial role in mesenchymal stem cell differentiation by promoting osteoblast differentiation [25] and in this process TAZ functions as a coactivator of Runx2, an osteoblast-specific transcription factor. Interestingly, *Runx2* and *TAZ* have been found over-expressed in thyroid papillary carcinoma [31], [32] and responsible for calcification processes present in these tumor tissues. Moreover, it has been recently demonstrated that Runx2 deficiency in mice causes decreased thyroglobulin expression [33]. All together, our data suggest that both *TAZ* and *Runx2* are direct targets of Pax8, thus it will be of great interest to investigate the effect of TAZ and Runx2 over-expression or silencing with respect to thyroid cells proliferation and differentiation.

*Trib1* is a member of a recently identified protein family called *tribbles*, that function as dynamic interactors of MAPK proteins, regulating numerous signaling pathways that play a role in embryonic development and the development of human diseases, such as cancer and autoimmune disease [34].

Finally, also involved in the regulation of cell proliferation there is the gene *Sparc*. Also named osteonectin or BM-40, it is a membrane-associated glycoprotein, which governs diverse cellular functions and has a pivotal role in regulating cell-matrix interactions, cellular proliferation and migration [35], [36]. Sparc plays as an important role in controlling malignancy of ovarian carcinoma [37] and during hypothalamic neuroendocrine cells development [38]. Recently, it has been demonstrated that *Sparc* is directly regulated by TTF-1/Nkx2.1 in Gonadotropin-Releasing Hormone (GRH) neurons [39]. TTF-1 (T-EBP/Nkx2.1) is a member of the Nkx-2 subfamily, originally identified as a protein able to bind to a DNA sequence that is present three times on both the Tg and the TPO promoters [40], [41]. We have, recently, demonstrated that the simultaneous expression of Pax8 and TTF-1 in thyroid cells has an important functional relevance since they strongly synergize in the transcriptional activation of thyroid-specific genes [8]. Interestingly, our data together with the already published evidences suggest that a cooperative regulation of Sparc expression by both transcription factors Pax8 and TTF-1 is likely to occur, strengthening the hypothesis of the existence of a transcriptional network that allows the fine-tuning of thyroid-specific gene expression involved in thyroid development and differentiation.

Among the genes belonging to the signal transduction category there is *Wnt4*. Initially classified as a non-canonical Wnt protein, it has been described to activate also the canonical signaling pathway. *Wnt4* knockdown underlined its crucial role in the development of several organ such as kidney, ovary, and mammary gland [42], [43], [44]. Mice lacking Wnt4 die after birth probably for the severe kidney dysfunction [43]. It has been demonstrated that Pax2, another member of the *Pax* gene family, is able to activate *Wnt4* promoter activity in a proximal tubule cell line promoting nephrogenesis [45]. At the same time, a cooperative role for Pax2 and Pax8 in metanephric branching morphogenesis and nephron differentiation has been uncovered [46]. Pax8 is required for the morphogenesis of the thyroid gland [7] and for the maintenance of the thyroid-differentiated phenotype [5]. Thus, it will be interesting to further analyze the potential function of Pax8/Wnt4 pathway during thyroid differentiation. In addition, the non-canonical pathway is crucial for the migration and also plays an important role in tumor biology [47]. Osamura group’s suggested that Wnt4/Fzd6 signaling is activated through the β-catenin-independent pathway in GHoma and TSHoma [48]. Together these findings suggest a potential involvement of Pax8/Wnt4 during the migration of the thyroid primordium and/or later on during development when the gland reaches its definitive position.

Some of the genes modulated by Pax8 belong to the protein and ion transport categories. The small GTPase, *Rab17*, a member of the rab family of regulators of intracellular transport, here identified as a novel Pax8 target, plays an important role in the control of transport through the apical recycling endosome and in the regulation of polarized membrane sorting [49].

*Kcnj16* (inward rectifier K+ channel subunit, also named *Kir5.1*) is involved in the regulation of fluid and pH balance. The analysis of *Kcnj16* ^−/−^ mice highlighted its crucial role in defining neuronal pH sensitivity [50]. Interestingly, our data indicated *Kcnj16* as a direct target of Pax8, suggesting that Pax8 may act on K+ channel level to regulate thyroid growth and function. At the same time, it is important to remember that proper thyroid function is guaranteed by the ability of the polarized epithelial cells to maintain the specific biochemical composition of the apical and basolateral plasma membrane domains.

Among the Pax8 novel targets unraveled in this study, we have identified the *Cdh-16* gene that belongs to the cell adhesion category. Cadherin-16, also named Ksp-cadherin, represents a structurally distinct member of the cadherins family. It belongs to a new subfamily of cadherins termed the 7D-cadherins, which are mainly characterized by two structural features: seven extracellular cadherin repeat domains (EC) and a highly truncated cytoplasmic tail. Identified as the only tissue-specific cadherin expressed exclusively in the kidney [51], it has been recently detected on the plasma membrane of human and mouse thyrocytes [52]. The long distance between the final location of the thyroid gland in front of trachea and the site of embryological specification at tongue base suggest that thyroid development could be influenced by cadherins, a superfamily of cell-cell adhesion molecules involved in migration, sorting, and re-aggregation of cells during embryogenesis [53]. Interestingly, the expression profile of *cadherin-16* resembles that of the transcription factor *Pax8*, making this adhesion molecule an excellent candidate to be a target of Pax8.

In this manuscript, we propose and discuss an overview of the genes potentially regulated by Pax8 in FRTL-5 cells inferred from the list of the differentially expressed genes in the microarray analysis. We identify potential Pax8 targets based on changes in transcripts levels, on the presence of Pax8 binding site(s) in their 5’-flanking sequences and tissue-specificity. We believe that our approach shows a great potentiality; in fact, we have been able to demonstrate that the majority (11 out of 17) of the genes that we have selected among the predicted Pax8 targets are indeed targets of this transcription factor. Therefore, it is likely that in the list of 276 genes that resulted from the PASTAA analysis there are many other interesting Pax8 targets to be further analyzed. Possibly, a more massive validation approach could take off a number of target genes almost comparable to a genome-wide ChIP-on-chip assay, which yet requires the availability of good specific antibodies.

Furthermore, this study shows that Pax8 is involved in the regulation of a large number of genes in differentiated FRTL-5 cells that will need to be evaluated by further studies to fully ascertain Pax8 direct targets. Although the broadness of the group of genes that could be regulated by Pax8 is consistent with its already known role of master regulator, it has highlighted several biological pathways downstream of this transcription factor that encourage further investigation of Pax8 function in new cellular processes.

## Materials and Methods

### Cell culture

The rat thyroid FRTL-5 cell line was used for this study (ATCC, cat. CRL-1468). FRTL-5 cells were grown in Coon’s modified F-12 medium (Euroclone) supplemented with 5% calf serum and a six-hormone mixture (6 H) as described by Ambesi-Impiombato and Coon [54].

### RNA interference

FRTL-5 cells were plated (8×10^4^ well) in 24-well plate and were transfected in triplicate with 100 nM Pax8 siGENOME siRNA or siGENOME Non-Targeting #3 (DHARMACON) as scramble using DharmaFECT 1 transfection reagent, following the manufacturer’s protocol. Sequences corresponding to the siRNA of Pax8 were sense, 5’-CCAUAUUAUUACAGCUCUA-3’; and antisense, 5’-UAGAGCUGUAAUAAUAUGG-3’. Cells were harvested 24 and 72 h after transfection and the total RNA was extracted.

### RNA extraction, quantitative real time RT–PCR and microarray hybridization

Total RNA was extracted using TRIzol reagent (Invitrogen) and treated with RNase-free DNase I (Applied Biosystem) for both qRT-PCR and microarray analyses.

For qRT-PCR the cDNA was synthesized using iScript cDNA Synthesis kit (BIORAD, Hercules, CA). Real time RT–PCR analysis was performed using IQ^TM^ SYBR Green PCR Master Mix (BIORAD) in an iCycler IQ^TM^ real-time detection system (BIORAD).

For microarray analysis, 5μg of total RNA extracted 72 h after transfection, were sent to the Affymetrix Microarray Unit at IFOM-IEO, Milan (http://www.cogentech.it) for labeling, amplification and hybridization to the AffymetrixGeneChip Rat Gene 1.0 ST array, according to manufacturer’s protocols. Three independent silencing experiments were performed from which three biological replicates of each condition (siPax8 or control) were processed for microarray analysis.

### Array data analysis

Array images have been preprocessed into CEL files using Affymetrix specific softwares. Gene-level signals were generated from CEL files with GeneSpring v.11.5 software (Agilent Technologies Inc.), using the Probe Logarithmic Intensity Error (PLIER) summarization algorithm, as recommended by Affymetrix protocols (http://www.affymetrix.com/support/technical/technotes/plier_technote.pdf).

After filtering genes with raw expression values lower than 20, differentially expressed genes, with a fold change higher than 1.2 in siPax8 vs. controls, were selected by one way ANOVA test and Benjamini and Hochberg false discovery rate (FDR) correction, using a 0.05 threshold for statistical significance.

All data is MIAME compliant and the raw data has been deposited in the MIAME compliant database Gene Expression Omnibus (GEO; http://www.ncbi.nlm.nih.gov/geo/) (accession n. GSE29341).

### Functional analysis

Gene ontology (GO) and pathway functional class scoring have been performed using the Gene Set Analysis Toolkit V2 (http://bioinfo.vanderbilt.edu/webgestalt) [55]. Using the Pathway-Express software (ONTO-tools, http://vortex.cs.wayne.edu/projects.htm) [56], the pathways most affected by the gene dysregulation were ranked according to the impact factor of the entire pathway, a probabilistic term that takes into consideration the proportion of differentially regulated genes in the pathway and therefore the pathway perturbation.

### Analysis of transcription factor binding sites (TFBSs)

The promoter regions of genes coregulated after Pax8 silencing were analyzed in order to recognize DNA binding motifs for both PAX8_01 and PAX8_B matrices (from TRANSFAC database). A strategy was developed to reduce the number of false positive targets filtering data according to tissue specificity and TFBSs conservation. Genes differentially expressed in siPax8 vs. controls were ranked according to their specific expression in thyroid tissue, using the BioGps gene annotation portal (http://biogps.gnf.org) [57] and the GeneHub Gepis bioinformatics tool (http://www.cgl.ucsf.edu/Research/genentech/genehub-gepis). Genes mostly expressed in either fetal or adult thyroid were selected for the TFBS analysis using the web-based PASTAA (predicting associated transcription factors from annotated affinities) method (http://trap.molgen.mpg.de) [17], which utilizes the prediction of binding affinities of a TF to promoters. The lists of downregulated and upregulated genes were ranked respectively according to the prediction of binding affinity of their promoter regions to the PAX8 binding sites, with the following criteria: range for promoter region from -10000 to 0 at either side of the transcription start site, conserved mouse/human sequence block, maximum affinity across promoter range.

### ChIP assay

Genomic sequences including 3 kb upstream of the start codon were retrieved from Genome website (http://www.genome.ucsc.edu) and were explored to predict potentially transcription factor binding sites using GEMS, Genomatix software (http://www.genomatix.de).

Chromatin immunoprecipitation (ChIP) was performed as follows. The cross-linking solution, containing 1% formaldehyde, was added directly to cell culture media. The fixation proceeded for 10 min and was stopped by the addition of glycine to a final concentration of 125 mM. FRTL-5 cells were rinsed twice with cold PBS plus 1 mM PMSF, and then scraped. Cells were collected by centrifugation at 800×g for min at 4 C. Cells were swelled in cold cell lysis buffer containing 5 mM piperazine-N,N-bis(2-ethanesulfonic acid) (pH 8.0), 85 mM KCl, 0.5% Nonidet P-40, 1 mM PMSF, and inhibitors cocktail (Sigma) and incubated on ice for 10 min. Nuclei were spun down by microcentrifugation at 2000×g for 5 min at 4 C, resuspended in nuclear lysis buffer containing 50 mM Tris-HCl (pH 8), 10 mM EDTA, 0.8% sodium dodecyl sulfate (SDS), 1 mM PMSF and inhibitors cocktail (Sigma), and then incubated on ice for 10 min. Samples were broken by sonication into chromatin fragments of an average length of 500/1000 bp and then microcentrifuged at 16,000×g. The sonicated cell supernatant was diluted 8-fold in ChIP Dilution Buffer containing 0.01% SDS, 1.1% Triton X-100, 1.2 mM EDTA, 16.7 mM Tris-HCl (pH 8.1), and 167 mM NaCl, and precleared by adding Salmon Sperm DNA/Protein A Agarose (Upstate Biotechnology, Inc., Lake Placid, NY) for 30 min at 4 C. Precleared chromatin from 1×10^6^ cells was incubated with 1 μg of affinity-purified rabbit polyclonal antibody anti-Pax8 (kindly provided by Prof. R. Di Lauro), or no antibody and rotated at 4 C for 16 h. Immunoprecipitates were washed five times with RIPA buffer containing 10 mM Tris-HCl (pH 8), 1 mM EDTA, 1% Triton X-100, 0.1% Na-deoxycholate, 0.1% SDS, 140 mM NaCl, and 1 mM PMSF; twice with LiCl buffer containing 0.25 M LiCl, 1% Nonidet P-40, 1% Na-deoxycholate, 1 mM EDTA, 10 mM Tris-HCl (pH 8.0), and then three times with TE (10 mM Tris-HCl, pH 8; 1 mM EDTA). Before the first wash, the supernatant from the reaction lacking primary antibody was saved as total input of chromatin and was processed with the eluted immunoprecipitates beginning at the cross-link reversal step. Immunoprecipitates were eluted by adding 1% SDS, 0.1 M NaHCO3 and reverse cross-linked by addition of NaCl to a final concentration of 200 mM and by heating at 65 C for 16 h. Recovered material was treated with proteinase K, extracted with phenol-chloroform-isoamyl alcohol (25∶24∶1) and precipitated. The pellets were resuspended in 30 μl of TE and analyzed by PCR using specific primers for the analyzed regions. The input sample was resuspended in 30 μl of TE and diluted 1∶10 before PCR.

## Supporting Information

Table S1 **The list of 2815 genes differentially expressed siPax8** **vs. controls with more than 1.2 absolute fold change and p <0.05.**
(XLS)

Table S2 **Lists of genes downregulated and upregulated by Pax8 silencing, that are mostly expressed in either fetal or adult thyroid, ranked according to the prediction of the affinity of their 5’-flanking regions to PAX8 consensus binding sequence.**
(XLS)

Table S3 **Oligonucleotide primers used in ChIP assays.**
(PDF)
