# Supplementary material for: Identification of Novel Pax8 Targets in FRTL-5 Thyroid Cells by Gene Silencing and Expression Microarray Analysis
Source: PLoS One. 2011 Sep 23;6(9):e25162. doi: 10.1371/journal.pone.0025162 (PMC3179481; doi:10.1371/journal.pone.0025162)
Supplement: Table S3 — Oligonucleotide primers used in ChIP assays. (PDF) [file pone.0025162.s003.pdf]

Supplemental Table 3. ChIP rat oligonucleotides list.

GENE NAME	FORWARD PRIMER 5'-3'	REVERSE PRIMER 5'-3'
Rab17	CCAGGATAGTCCCTCTCTCC	AGCTCAAGCCTCAGAGCACC
Trib1	CTCGATCCCCACAACCTGTC	CTACCGGAAGCAGTCAAGCTC
Wnt4	GATCCAGAAGCGAGGTTTCGG	AACCTAGTCACTAGCGCTCGG
Tg	ATGTCCTGGAGTGGTCACCC	GGGTAGGAGCAGTGTCCCATC
Kcnj16	GATGAAGGTCCACAGGAACCC	GTGACCAACAGCTGCAGCAAC
Cdh16	AGAAGTGGGGCCAAGTCTGAAGCC	GGGGCGAGGCAAGGTGGACACTT
Foxe1	CAGCGGAGGGAGGAGCTG	CATGCCGCACCTCTGCGG
Runx2	CTACGGAACTGGAACCGGGA	TTTGTCCCAGCCGAGGCAAG
Cited2	GAAAACTCTGCTGGGCAGGG	CGGACTGTGGTGTCTTTCTGG
TAZ	GAATGCAGATAGAGCGCTCGG	GGCCGAAAGTGGAGCTGTTG
Sparc	GATGAGGACGATGCCACCAG	TCCTGGGCATTACCCGAGAG
